# Comparison of different surface disinfection treatments of drinking water facilities from a corrosion and environmental perspective

**DOI:** 10.1007/s11356-020-07801-9

**Published:** 2020-02-01

**Authors:** Valentin Romanovski, Per Martin Claesson, Yolanda Susanne Hedberg

**Affiliations:** 1 Department of Chemistry, School of Engineering Sciences in Chemistry, Biotechnology and Health, Division of Surface and Corrosion Science, KTH Royal Institute of Technology, SE-100 44 Stockholm, Sweden; 2 Center of Functional Nano-Ceramics, National University of Science and Technology “MISIS”, Moscow, Russia 119049; 3 Institute of General and Inorganic Chemistry, National Academy of Sciences of Belarus, 220072 Minsk, Belarus; 4 RISE Research Institutes of Sweden, Bioscience and Materials – Surface, Process and Formulation, SE-114 86 Stockholm, Sweden

**Keywords:** Surface disinfection, Water facility, Groundwater, Corrosion, Life cycle assessment, Environmental impact

## Abstract

Surface disinfection of water facilities such as water wells requires measures that can remove pathogens from the walls to ensure a high drinking water quality, but many of these measures might increase corrosion of the contact surfaces (often highly pure steel) and affect the environment negatively due to disinfectant-contaminated waste sludge and wastewater. Today, most treatments worldwide are based on hypochlorites. We investigated the extent of corrosion during treatments of steel at relevant conditions of ozone, sodium, and calcium hypochlorite for drinking water preparation, utilizing weight loss, electrochemical, solution analytical, and surface analytical methods. The ozone treatment caused significantly less corrosion as compared with sodium or calcium hypochlorite with 150–250 mg/L active chlorine. Hypochlorite or other chlorine-containing compounds were trapped in corrosion products after the surface disinfection treatment with hypochlorite, and this risked influencing subsequent corrosion after the surface disinfection treatment. A life cycle impact assessment suggested ozone treatment to have the lowest negative effects on human health, ecosystems, and resources. Calcium hypochlorite showed the highest negative environmental impact due to its production phase. Our study suggests that ozone surface disinfection treatments are preferable as compared with hypochlorite treatments from corrosion, economic, and environmental perspectives.

**Electronic supplementary material:**

The online version of this article (10.1007/s11356-020-07801-9) contains supplementary material, which is available to authorized users.

## Introduction

Drinking water facilities are regularly decontaminated by surface disinfection treatments to inactivate pathogens and microorganisms. This disinfection of internal surface of water supply facilities should be carried out at least once a year, and additionally in the case of viruses and microorganisms in the water. This is for example accomplished by chlorine-containing reagents, such as sodium and calcium hypochlorite (American Water Works Association 2013, Jackson et al. 2001, Salvato 1992). The treatment with chlorine-containing substances is used worldwide. As an alternative, ozone was suggested for surface disinfection of water facilities (Schulz & Lohman 2005), where the surfaces to be decontaminated are often made of low-alloyed steel. This treatment has been used and studied for stainless steels and in the food industry before (Greene et al. 1993, Guzel-Seydim et al. 2004), and was found to be beneficial as compared with chlorine-based disinfectants from an environmental perspective (Pascual et al. 2007). It has also been used for the drinking water preparation in some countries (Geering 1999, Rakness 2011), while others totally rely on chlorine-based disinfection. One important aspect when choosing surface disinfection treatment is the short- and long-term corrosion expected by the different surface disinfection treatments. Studies comparing different surface disinfection treatments for low-alloyed steel used in the preparation of drinking water in water wells are rare.

Regular surface disinfection treatments are a necessity, but some of these, or their by-products, may be hazardous for the environment and health (Bull 1982, Jeong et al. 2012, Villanueva et al. 2003). One example is disposal of chlorine-containing sewage that is collected after treatment, which can be hazardous to the environment (Bayo et al. 2009). The disinfection also results in accelerated material degradation. This may lead to increased corrosion even after the treatment period, if disinfectants would be trapped in pores or cracks. Treatment times are usually 6–24 h for chlorine-based treatments, but only 0.5 h for ozone treatment (Романовский et al. 2016).

The current amount of sodium hypochlorite used in the European Union is in the range of 1,000,000–10,000,000 tons per annum (ECHA 2019b), while the registered amount of calcium hypochlorite is 0–10 tons per annum (ECHA 2019c). Sodium hypochlorite is currently classified as “H400 Very toxic to aquatic life” and “H410 Very toxic to aquatic life with long-lasting effects” by the European Chemical Agency (ECHA 2016). Calcium hypochlorite is also classified as “H400 Very toxic to aquatic life” (ECHA 2016). On a consumption basis, sodium hypochlorite accounted for 91% of the total global hypochlorite use, with calcium hypochlorite making up the rest (IHS Markit 2015). Consumption of sodium hypochlorite in laundry bleach applications currently accounts for 67% of its use, with disinfectant use accounting for the remaining 33% (IHS Markit 2015). Thus, the use of hypochlorite is significant, and a change to less harmful chemicals, such as ozone (Bhuvaneshwari et al. 2019, Dong et al. 2018, Sun et al. 2018), could result in environmental and health benefits. Comparative quantitative and qualitative data on corrosion induced by hypochlorite and ozone surface disinfection treatments would in addition provide knowledge on technological, economic, and societal consequences of different surface disinfectant treatments for low-alloyed steel in water well facilities for the preparation of municipal drinking water.

The objectives of this study were (i) to compare low-alloyed steel corrosion for relevant surface disinfection treatments using hypochlorite-based and ozone-based methods, (ii) to estimate any increased subsequent corrosion after surface disinfection treatment, and (iii) to provide a life cycle impact assessment of the different surface disinfection treatments. Note that this study is devoted to the disinfection of the internal surface of water supply facilities (water boreholes, filters, pipes, reservoirs) and not to disinfection of water volume on the way to households, which would require much lower concentrations of disinfectants.

## Materials and methods

### Materials and sample preparation

The material used in the present work was steel for water well pipes (standard: EN 10130/91; quality: Fe P01 A). Its nominal composition (wt%), iron (balance), 0.06 wt% C, 0.22 wt% Mn, 0.01 wt% Si, 0.014 wt% P, 0.014 wt% S, 0.047 wt% Al, and 0.006 wt% N, is similar to that analyzed in steel samples from different water facilities (Table S1, supplementary information).

Steel samples with a thickness of 1.5 mm and dimensions of 15 × 15 mm were abraded with emery papers down to FEPA P 1200# SiC grit and then polished using short synthetic DP-Nap and diamond pastes (down to 1 μm) DP-Stick P Struers. The samples were thereafter washed with ultrapure water (18.2 MΩ cm, Milli-Q system, Millipore, Sweden), degreased ultrasonically by acetone and ethanol (for 5 min each), and dried with nitrogen gas at room temperature (≈ 21 °C). All steel samples were then kept in a desiccator at room temperature for 24 ± 2 h prior to testing.

### Solutions and disinfection agents

Experiments were carried out in solutions based on artificial tap water (ISO 6341:^2012^ 2012). The composition of the artificial tap water was as follows: 65.7 mg/L NaHCO_3_, 5.75 mg/L KCl, 123.0 mg/L MgSO_4_·7H_2_O, 294.0 mg/L CaCl_2_·2H_2_O, pH 7.5 (adjusted by 1% HNO_3_). The disinfectants used were NaClO (initial concentration 3.5 g/L of active chlorine, VWR chemicals, France) and Ca(ClO)_2_ (99.95%, initial concentration 65% of active chlorine, Alfa Aesar, Germany). The corrosion experiments were performed in aqueous solutions of hypochlorite with 50, 150, and 250 mg/L of active chlorine. The active chlorine or free chlorine is known as a hypochlorite ion OCl^−^ concentration (Brandt et al. 2016). The concentrations used are relevant for disinfection of water treatment facilities (American Water Works Association 2013, Jackson et al. 2001, Salvato 1992).

Ozone was generated by utilizing an Internal ozone generator (Model O341M Environment S.A). The ozone concentration in the outlet was 0.75 mg/m^3^ and the flow rate was 4 L/min, resulting in an ozone concentration of 0.36 mg/L in a 250 mL cell after 30 min treatment, which is a relevant concentration for surface disinfection (World Health Organization 2011). All chemicals used in this study were of p.a. or puriss. p.a. grade (in the case of metal release investigations). The solvent for all solutions was ultrapure water.

### Surface morphology and composition

The surface morphology and surface composition (with μm-resolution) were determined prior to and after treatment using a tabletop scanning electron microscope (SEM) with backscattered electron analysis (Hitachi TM-1000) coupled to energy X-ray dispersive spectroscopy (EDS). The microstructure and chemical composition of the scratched-off corrosion products were studied by SEM using a JEOL F7600 equipped with backscattered electrons (BSE) and EDS. At least two samples were investigated for each condition. The surface analyses were conducted with magnifications up to × 20,000 for a minimum of 10 different locations.

### Raman spectroscopy

A Horiba HR800 instrument was used for confocal Raman spectroscopy measurements, using a 514-nm laser (no filter), a 500-μm pinhole, and an Olympus × 50 (0.25 NA) objective. The sample was inspected before and after analysis to ensure no laser-induced oxidation.

### Corrosion estimations and electrochemical measurements

Weight loss measurements were carried out for up to 24 h of treatment in closed glass flasks in artificial tap water, artificial tap water containing NaClO or Ca(ClO)_2_, or in artificial tap water treated with ozone during the first 30 min. The exposed surface area of the samples was 3.9 or 4.7 cm^2^ in a 10-mL solution. The samples were taken out from the solutions after 6, 12, 18, and 24 h, rinsed by 2 wt% citric acid solution (during about 10 s) in order to remove the corrosion products formed (Varga et al. 2001), rinsed by deionized water, and dried by nitrogen gas. This procedure did visually remove corrosion products without attacking the metal. The samples were then weighed with 0.1 mg accuracy using a balance (Mettler Toledo), and the weight was compared with their initial weight. All weight loss measurements were replicated on three parallel samples. Both the treatment solution and the citric acid solution used for removal of corrosion products were analyzed to determine their iron concentration, as described in the next section.

Electrochemical measurements consisted of open-circuit potential (OCP) and potentiodynamic polarization scans. The multichannel Princeton Applied Research AMETEK potentiostat was used with three-electrode cells (250 mL) using a KCl saturated Ag/AgCl reference electrode, a Pt-mesh counter electrode, and the steel sample as the working electrode. The exposed area of the sample was 0.785 cm^2^. All measurements were performed at room temperature (≈ 21 °C) and replicated at least three times. The OCP curves were measured each second during 24 h. The potentiodynamic curves were obtained in the potential range − 0.25 V to + 1.0 V (vs. OCP, which was first determined during 5 min) with a scan rate of 0.5 mV/s. Electrochemical parameters were extracted from the polarization curves using the Levenburg-Marquardt method (Gavin 2019) in the VersaStudio software (2017).

### Iron determination in solutions

Solution metal analysis was performed using atomic absorption spectroscopy (AAS, AAnalyst 800 instrument, Perkin Elmer) in the flame mode. Calibration curves were based on at least four calibration standards, which were covering the sample concentrations, and quality control samples of known concentration were analyzed regularly. The limits of detection, as determined by three times the highest standard deviation of the blank samples, were approximately 0.2–1 mg/L, depending on the calibration standards used. All sample concentrations exceeded the limits of detection and were higher than in the corresponding blank (background solutions without any contact with steel). The reported concentration of iron in solution is based on the average concentration of three independent replicate samples with the corresponding blank concentration subtracted. The error bars in the figures show the standard deviation of three independent samples. For all samples, the aqueous iron concentration immediately after treatment and the iron concentrations resulting from dissolving the rust with citric acid and nitric acid were determined (Fig. S1 (supplementary information)). The rust that could not be dissolved by citric acid and nitric acid was examined by EDS.

### Chemical speciation modeling

The Joint Expert Speciation System (JESS, (May 2015)) was used to model the chemical speciation of iron species in the different solutions. As input values, 1.8 mM Fe^3+^, a temperature of 25 °C, atmospheric pressure, 0.782 mM CO_3_^2−^, 0.0771 mM K^+^, 0.499 mM SO_4_^2−^, and 0.499 mM Mg^2+^ were used for all cases, while the concentrations of Na^+^, Ca^2+^, Cl^−^, the pH, and the redox potential were varied as shown in Table 1. The redox potential was increased for increasing hypochlorite concentration in order to simulate the increasing oxidizing potential. Precipitation of solid species was allowed (and investigated). Thirty-five different solids of different iron compounds were identified and included by the chemical speciation system.

**Table 1:** Varying input values in JESS modeling for the different solutions investigated. *a.ch.*, active chlorine

Solution	Na^+^ (mM)	Ca^2+^ (mM)	Cl^−^ (mM)	pH	Redox potential (pe)
Artificial tap water (ATW)	0.782	2.00	4.08	7.5	5
ATW + 50 mg/L a.ch. NaClO	5.83	2.00	5.05	9; 8.3	6
ATW + 150 mg/L a.ch. NaClO	7.77	2.00	6.99	9.7; 8.5	8
ATW + 250 mg/L a.ch. NaClO	9.72	2.00	8.94	10; 8.6	10
ATW + 50 mg/L a.ch. Ca(ClO)_2_	0.782	4.52	5.05	8.5; 8.2	6
ATW + 150 mg/L a.ch. Ca(ClO)_2_	0.782	5.50	6.99	8.6; 8.2	8
ATW + 250 mg/L a.ch. Ca(ClO)_2_	0.782	6.47	8.94	8.7; 8.2	10

### Life cycle analysis and life cycle cost analysis

The software SimaPro 8.04.26 including the research method ReCiPe Endpoint (I) V1.11/Europe ReCiPe I/A was used to assess the impact of the surface disinfectants on the environment during their life cycle. The evaluation method ReCiPe Endpoint (I) has three endpoints: damage to human health, ecosystem quality, and damage to resource (Goedkoop et al. 2009, Sleeswijk et al. 2008). The basic unit of overall environmental impacts was Pt (point-standard eco-indicator normalized unit). In LCA, for the method ReCiPe Endpoint (I), 17 different impact categories (midpoints) were analyzed in detail, as human toxicity was split up into “carcinogens” and “non-carcinogens.” Baseline data for the calculation was taken from the preparation conditions of 1 m^3^ of disinfectant solution with concentration 150 mg active chlorine/L (or 1 mg/L ozone). The efficiency of the use of raw materials in the preparation of reagents was assumed to be 90%. Further details are given in the supplementary material (Figs. S2-S5, Tables S2-S4).

### Statistical significance

Statistically significant differences between sample data sets were estimated by Student’s *t* test for unpaired data of unequal variance in the KaleidaGraph v.4.0 software.

## Results

When evaluating the corrosion behavior, we considered relevant treatment times needed for surface disinfection by hypochlorites (typically 12–24 h) and by ozone (typically 30 min) (Романовский et al. 2016).

**Fig. 1:**
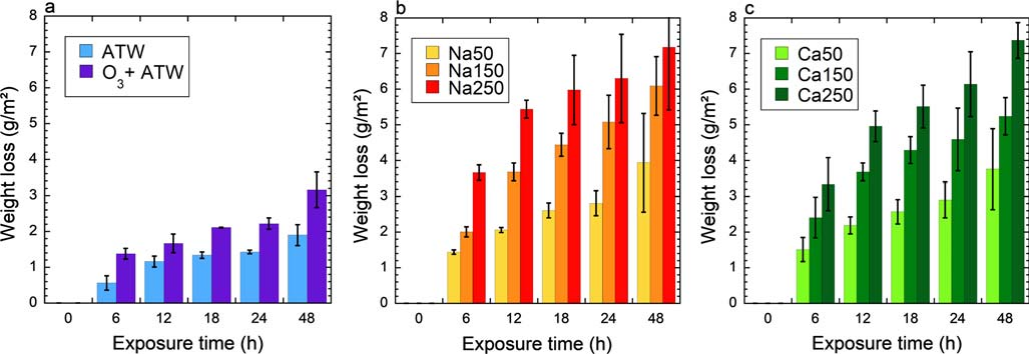
Accumulative weight loss (g/m^2^) of steel in selected solutions: artificial tap water (ATW), and ozone treatment during the first 30 min followed by fresh artificial tap water for up to 48 h (**a**); artificial tap water with 50–150 mg/L active chlorine of NaClO (**b**) or Ca(ClO)_2_ (**c**) during 24 h, followed by fresh artificial tap water for 24 h

Weight loss experiments revealed a decreasing corrosion rate with time for all conditions tested. The weight loss of the samples was similar in sodium and calcium hypochlorite, and increased with increasing hypochlorite concentration (Fig. 1). The weight loss in ozonated artificial tap water was comparable or slightly lower than the weight loss of the samples in sodium and calcium hypochlorite at their lowest concentration of 50 mg/L active chlorine. After 24 h, the weight loss of steel in ozonated water was 2.1 g/m^2^, which is a factor of 2–3 less than in 150 mg/L active chlorine of sodium and calcium hypochlorite, and 20–30% less than in 50 mg/L active chlorine. The corrosion rate of steel in 30 min ozonated artificial tap water was 1.5-fold higher than in non-ozonated water.

Next, we determined the effect of previous disinfection treatments on corrosion of the steel samples. After the initial disinfection treatment for 24 h (NaClO, Ca(ClO)_2_, or ozone during the first 30 min followed by no additional ozone during 23.5 h), all samples were taken out from the treatment solution, washed by ultrapure water, and immersed into fresh artificial tap water for 24 h. The last time point of 48 h in Fig. 1 shows this subsequent artificial tap water exposure. The weight loss increased further in all cases, however at a significantly lower rate as compared with the initial 24 h (Fig. 1).

**Fig. 2:**
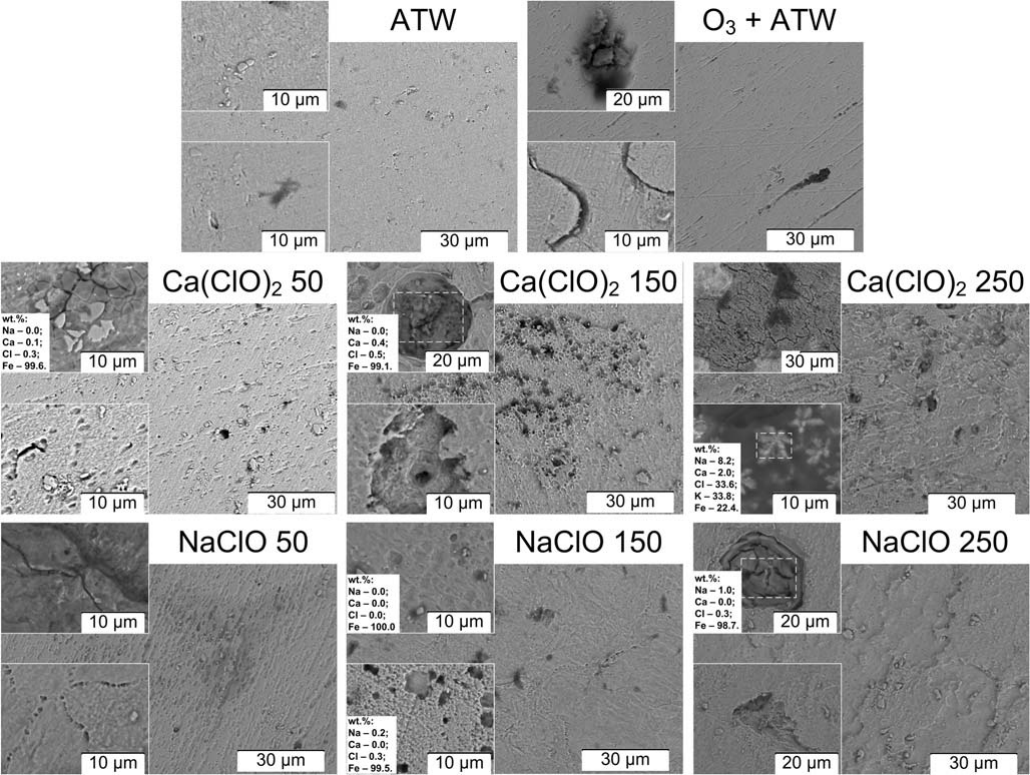
Scanning electron microscopy images (× 2000 magnification) of the surface of steel after 24 h of treatment (rinsed with ultrapure water). Treatments: ATW (artificial tap water), O_3_ + ATW: 30 min ozone treatment followed by 23.5 h continued exposure in artificial tap water without ozone, Ca(ClO)_2_ or NaClO treatments of 50, 150, or 250 mg/L active chlorine in artificial tap water for 24 h. Mass concentrations measured by energy dispersive X-ray spectroscopy are indicated for selected samples

SEM images clearly reflect the weight loss measurements, that is, the lowest extent of corrosion in artificial tap water, followed by ozone treatment, and the hypochlorite solution treatments, which show a larger extent of corrosion with increasing active chlorine concentrations (Fig. 2). Despite rinsing with ultrapure water after treatment, the hypochlorite solutions seemed to influence the extent and composition of the corrosion deposit (Fig. 2). Sodium was detected in corrosion products of the NaClO-treated samples to a greater extent as compared with the other treated samples, and calcium was detected to a greater extent on the samples previously treated with Ca(ClO)_2_. Chlorine was only detected for the samples previously treated with hypochlorite solutions. Note that all samples have been treated in artificial tap water, which contains small amounts of, among others, calcium, sodium, potassium, and chlorides. Cracks and local defects were visible in all surface disinfection treated samples.

**Fig. 3:**
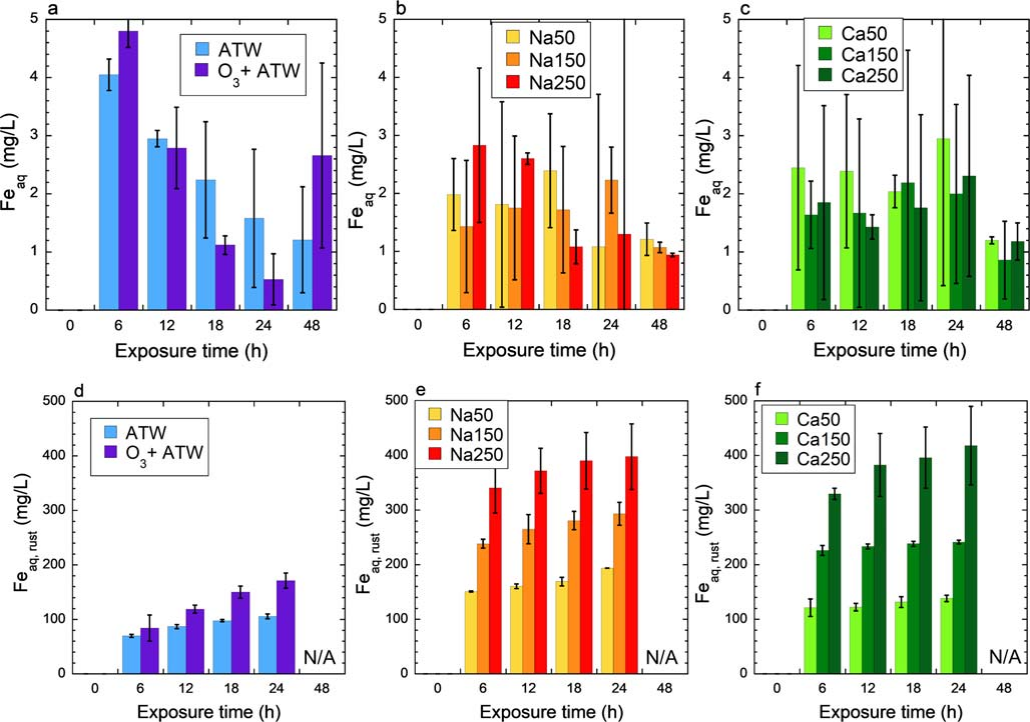
Iron in solution (Fe_aq_) after treatments (**a**–**c**) and from corrosion products after treatments rinsed by citric acid and dissolved in nitric acid (**d**, **e**, Fe_aq, rust_). Treatments: ATW (artificial tap water), O_3_ + ATW: 30 min ozone treatment followed by 23.5 h continued exposure in artificial tap water without ozone (**a**, **d**), NaClO (**b**, **e**) or Ca(ClO)_2_ (**c**, **f**) treatments of 50, 150, or 250 mg/L active chlorine in artificial tap water for 24 h. These treatments were followed by fresh ATW for another 24 h (denoted 48 h) (**a**–**c**). N/A, not analyzed

The dissolved iron concentration after immersion of the steel samples in different solutions was determined by AAS analysis directly after treatment and the acidification by nitric acid to a pH less than 2. In general, the iron concentration was relatively low, a few mg/L. There was a clear decreasing trend with time for artificial tap water and for the ozone treatment, whereas a similar but less clear trend was observed for hypochlorite treatment. This is as expected for thermodynamically unstable aqueous iron at pH values exceeding pH 7.5 (Beverskog & Puigdomenech 1996) (Fig. 3a). Artificial tap water and ozone-treated artificial tap water seemed to keep a greater extent of iron in solution as compared with the more alkaline hypochlorite solutions (Fig. 3a–c). Chemical speciation modeling results are presented in Table 2. The second (lower) pH value for the hypochlorite solution represents the final measured pH value after 24 h. It is clear from Table 2 and Fig. 3 that the theoretical solubility of iron in artificial tap water is even lower than that measured after 48 h of exposure in artificial tap water, which explains the decreasing concentrations measured over time. The measured concentrations of iron in the hypochlorite solutions after 24 h are slightly higher (1–2 mg/L iron) than those predicted in solution (about 1 mg/L, Table 2). The opposite is the case for the sodium hypochlorite solution at high active chlorine (150–250 mg/L) and therefore high pH values, predicting about 4–6.5 mg/L iron as FeOH_5_^2−^ in solution, while 1–3 mg/L iron were analyzed. The large error bars may reflect on-going precipitation processes resulting in high variance among independent samples.

**Table 2:** Predominating species as calculated by JESS (input values in Table 1 and “Chemical speciation modeling”). pH values represent the measured pH values (in the case of hypochlorite solutions showing first the initial and then the final pH). *a.ch.*, active chlorine; *s*, solid; *aq*, aqueous

Solution	Predominating species
Artificial tap water (ATW)	pH 7.5: 100 mg/L (100%) α-Fe_2_O_3_ (s), 36 μg/L (0%) FeOH_5_^2−^ (aq)
ATW + 50 mg/L a.ch. NaClO	pH 9: 61 mg/L (61%) α-Fe_2_O_3_ (s), 39 mg/L (39%) FeOH_5_^2−^ (aq). pH 8.3: 99 mg/L (98%) α-Fe_2_O_3_ (s), 1.5 mg/L (2%) FeOH_5_^2−^ (aq).
ATW + 150 mg/L a.ch. NaClO	pH 9.7: 101 mg/L (100%) FeOH_5_^2−^ (aq). pH 8.5: 97 mg/L (96%) α-Fe_2_O_3_ (s), 4.0 mg/L (4%) FeOH_5_^2−^ (aq).
ATW + 250 mg/L a.ch. NaClO	pH 10: 101 mg/L (100%) FeOH_5_^2−^ (aq). pH 8.6: 94 mg/L (93%) α-Fe_2_O_3_ (s), 6.5 mg/L (7%) FeOH_5_^2−^ (aq).
ATW + 50 mg/L a.ch. Ca(ClO)_2_	pH 8.5: 97 mg/L (96%) α-Fe_2_O_3_ (s), 4.0 mg/L (4%) FeOH_5_^2−^ (aq). pH 8.2: 100 mg/L (99%) α-Fe_2_O_3_ (s), 1.0 mg/L (1%) FeOH_5_^2−^ (aq).
ATW + 150 mg/L a.ch. Ca(ClO)_2_	pH 8.6: 94 mg/L (93%) α-Fe_2_O_3_ (s), 6.7 mg/L (7%) FeOH_5_^2−^ (aq). pH 8.2: 99 mg/L (99%) α-Fe_2_O_3_ (s), 1.1 mg/L (1%) FeOH_5_^2−^ (aq).
ATW + 250 mg/L a.ch. Ca(ClO)_2_	pH 8.7: 89 mg/L (89%) α-Fe_2_O_3_ (s), 11 mg/L (11%) FeOH_5_^2−^ (aq). pH 8.2: 99 mg/L (99%) α-Fe_2_O_3_ (s), 1.1 mg/L (1%) FeOH_5_^2−^ (aq).

A citric acid rinsing procedure was used to remove solid corrosion products, followed by further acidifying in nitric acid. Most of the corrosion products were dissolved in citric and nitric acid, and the soluble iron in solution was only 1–5% of the iron in citric and nitric acid dissolved corrosion products (Fig. 3). The amount of dissolved iron from corrosion products follows the trends observed with weight loss measurements (Fig. 3). The corrosion products were however not completely soluble in citric acid and nitric acid, which was especially evident for longer exposure times (Fig. S6 (supplementary information)). For the shortest exposure time point, 6 h, all corrosion products of artificial tap water–treated steel were soluble in citric and nitric acid, but for steel treated in 50 mg/L active chlorine Ca(ClO)_2_ for 24 h, 70% of the corrosion products were insoluble (Fig. S6), as determined from the difference in weight loss and measured iron in solution (corrosion products dissolved by citric and nitric acids and released iron).

EDS analysis of the insoluble corrosion products revealed the presence of calcium and iron for corrosion products obtained from both sodium and calcium hypochlorite treatments (Fig. S7 (supplementary material)). This demonstrates that a calcium- and iron-containing corrosion product is formed during prolonged treatment times, and that it is insoluble in citric and nitric acid.

**Fig. 4:**
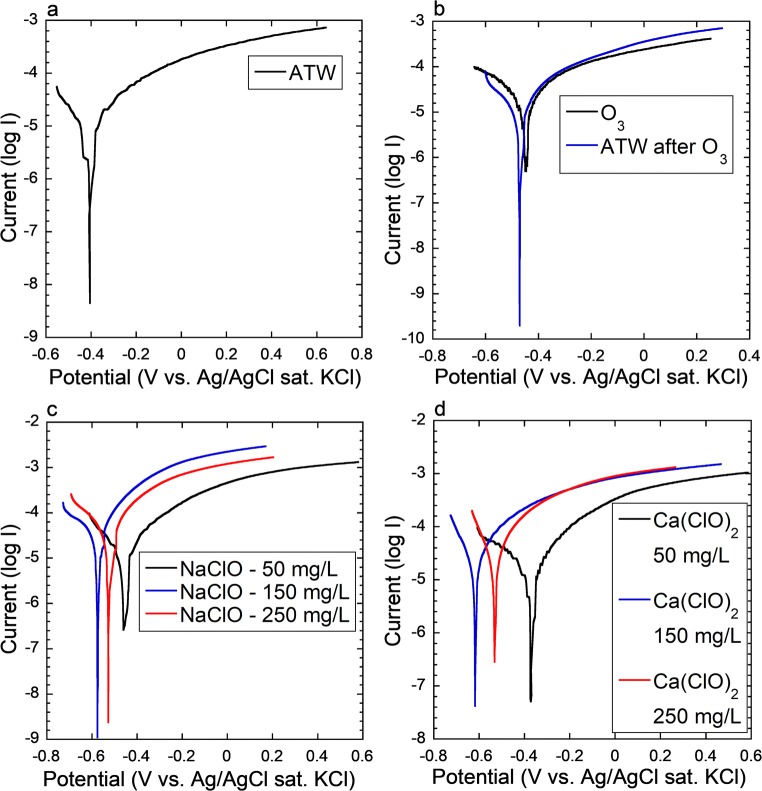
Potentiodynamic polarization curves (scan rate 0.5 mV/s scanning in anodic direction) in artificial tap water (ATW, **a**), during ozone treatment and after ozone treatment of 30 min (**b**), and in different hypochlorite solutions with active chlorine concentrations of 50–250 mg/L (**c**, **d**). Representative curves of two measurements are shown. All measurements were conducted after 5 min of open circuit potential

The potentiodynamic curves for the steels in selected solutions of artificial tap water, artificial tap water during and after injection of ozone, and sodium and calcium hypochlorite solutions are shown in Fig. 4. The corresponding electrochemical parameters extracted from the polarization curves are summarized in Table 3.

**Table 3:** Electrochemical parameters extracted from potentiodynamic polarization measurements. Average and standard deviations of two independent measurements are shown

Solution	E_corr_, mV	i_corr_, μA/cm^2^	Cathodic β, mV	Anodic β, mV
Artificial tap water	− 401.6 ± 4.1	6.7 ± 0.6	144.4 ± 4.0	87.3 ± 5.2
NaClO 50 mg a.ch./L	− 456.1 ± 5.1	19.1 ± 1.1	210.3 ± 40.8	173.9 ± 60.5
NaClO 150 mg a.ch./L	− 591.5 ± 15.2	20.3 ± 5.6	108.5 ± 19.5	104.4 ± 13.4
NaClO 250 mg a.ch./L	− 524.6 ± 7.4	25.5 ± 3.5	104.6 ± 7.6	101.4 ± 15.0
Ca(ClO)_2_ 50 mg a.ch./L	− 387.2 ± 22.4	17.5 ± 3.2	204.6 ± 20.3	146.4 ± 21.5
Ca(ClO)_2_ 150 mg a.ch./L	− 589.4 ± 43.4	19.5 ± 1.5	120.1 ± 25.0	122.1 ± 16.6
Ca(ClO)_2_ 250 mg a.ch./L	−548.6 ± 20.6	21.1 ± 1.1	102.0 ± 31.6	100.6 ± 18.4
O_3_ during 30 min treatment	− 445.9 ± 0.04	10.0 ± 1.1	102.1 ± 5.6	92.6 ± 8.5
after 30 min treatment of O_3_	− 469.0 ± 2.2	7.1 ± 1.8	115.0 ± 7.0	96.4 ± 7.7

*E*_*corr*_, corrosion potential; *i*_*corr*_, corrosion current density; *a.ch.*, active chlorine; hypochlorite- and ozone-treated solutions in artificial tap water

**Fig. 5:**
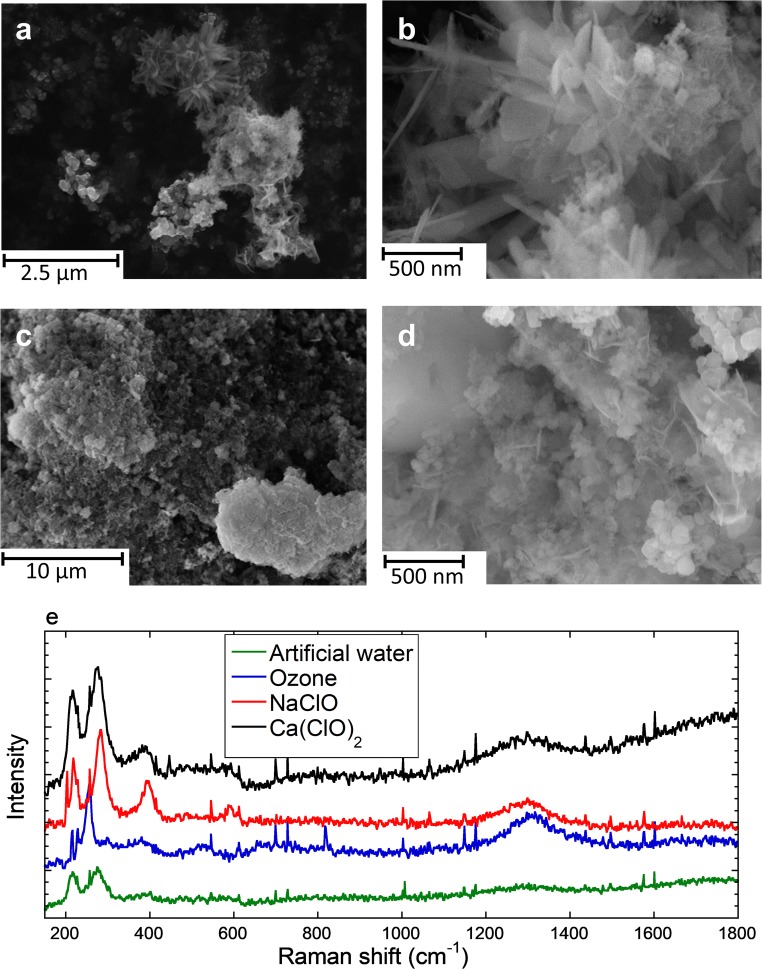
Scanning electron microscopy images of corrosion products for the corrosion products obtained after 24 h of treatment in sodium (**a**, **b**) and calcium (**c**, **d**) hypochlorite in artificial tap water (150 mg/L active chlorine). **e** Average (of two independent samples acquired for 180 s each) Raman spectra for the corrosion products obtained after 24 h of treatment in artificial tap water, ozone in artificial tap water (30 min followed by 23.5 h without ozone), and hypochlorite in artificial tap water (150 mg/L active chlorine). The spectra are off-set for clarity. Single spectra are shown in Fig. S11 (supplementary material)

Hypochlorite treatment resulted in the highest corrosion current densities, with the highest corrosion current densities at 150 mg/L active chlorine (Table 3 and Fig. 4). The corrosion current density in artificial tap water with 150 mg/L of active chlorine was 4.1 and 3.9 times higher for sodium and calcium hypochlorite solutions compared with artificial tap water. Two hundred fifty milligrams per liter active chlorine hypochlorite solutions showed instead a slightly lower corrosion current density as compared with 150 mg/L solutions, most probably related to a more rapid passivation or a higher pH value (see pH values reported Fig. 5). The corrosion current density during the ozone injection was a 2-fold higher compared with artificial tap water. Cessation of the ozone injection resulted in a reduction of the corrosion current density by 30%. From the corrosion current density, the weight loss can be calculated by Faraday’s law, as shown in the supplementary material. Figures S8 and S9 (supplementary material) show that this calculated weight loss was equal or larger as compared with the weight loss measured directly after 6 h of exposure (Fig. 1). The larger weight loss estimated from the electrochemical measurements is probably an effect of linear extrapolation from the electrochemical measurements that were conducted during 15 min as compared with the weight loss after 6 h.

Open-circuit potential over time was also measured for 24 h for the different surface disinfectant treatment conditions (Fig. S10 (supplementary material)). For all solutions, the open-circuit potential first decreased with time and then stabilized at a low value, showing an active corrosion behavior. The stabilization time was found to depend on the solution composition and pH. For artificial tap water solutions of different pH values, an increased pH resulted in a longer stabilization time and a higher stabilization open-circuit potential. The pH seemed to be the main factor influencing the stabilization time and stabilization potential in all solutions (Fig. S10), with the exception of the high active chlorine-containing solutions (Fig. S10c, e) that have a shorter stabilization time than what would be expected from their pH value alone (Fig. S10a). After the treatment for 24 h in chlorine-containing disinfectants, the loosely attached corrosion products were rinsed off from the samples with ultrapure water and their OCP was measured for the next 24 h in artificial tap water. The leaching of hypochlorite from pores increased the pH of the artificial tap water for the samples that previously had been treated by hypochlorite (Figs. S10d and S10f).

After the 24 h treatments, the corrosion products were mechanically removed and dried in ambient air. They were then analyzed by means of SEM, EDS, and Raman spectroscopy (Fig. 5 and Table S5 (supplementary material)). The morphology of corrosion products after the sodium (Figs. 5a, b) and calcium (Fig. 5c, d) hypochlorite treatments was slightly different. Both needle-shaped, platelets, and spherical particles were found, without any obvious difference in composition measured by EDS mapping, with one exception (the large particle to the top left in Fig. 5d is calcium-rich and iron-depleted). There was however a clear difference in composition for the sodium compared with calcium hypochlorite–treated steel corrosion products: sodium was only present in sodium hypochlorite–treated corrosion products, and calcium was (to a lower extent) only present in calcium hypochlorite–treated corrosion products (Table S5). The atomic ratio between iron and oxygen (oxygen contains contributions from other oxides and organic atmospheric contaminants) was 0.36 ± 0.20 for sodium and 0.38 ± 0.11 for calcium hypochlorite–treated steel corrosion products. Clear Raman peaks were found at 210–220 cm^−1^ in all cases (Fig. 5e). A broader peak at 250–290 cm^−1^ was found for artificial tap water– and Ca(ClO)_2_-treated steels. Ozone-treated steel revealed a narrower peak at 250 cm^−1^ and NaClO-treated steel at 285 cm^−1^. These peaks, as well as the peak at 1300 cm^−1^ present for all samples, indicate hematite (α-Fe_2_O_3_) (De Faria et al. 1997). For the ozone-treated sample, small amounts of lepidocrocite (γ-FeO(OH)) could also be present, showing peaks at 250, 370, 500 (shoulder), 520, 650 (shoulder), 720, and 1300 cm^−1^ (De Faria et al. 1997). For both hypochlorite solutions, an additional clear peak at around 380–390 cm^−1^ is present. This is most probably related to the Fe-Cl band (Réguer et al. 2007), either from FeCl_3_, which is characterized by two peaks at 400 and 600 cm^−1^ (Zhou et al. 2017), or from akaganéite (β-FeO(OH, Cl)), which has main peaks at 307 and 387 cm^−1^ (Réguer et al. 2007), or a combination of these. Both EDS and Raman spectroscopy analyses suggest the presence of chlorine- and iron-containing corrosion products in the case of treatment with hypochlorite solutions.

**Fig. 6:**
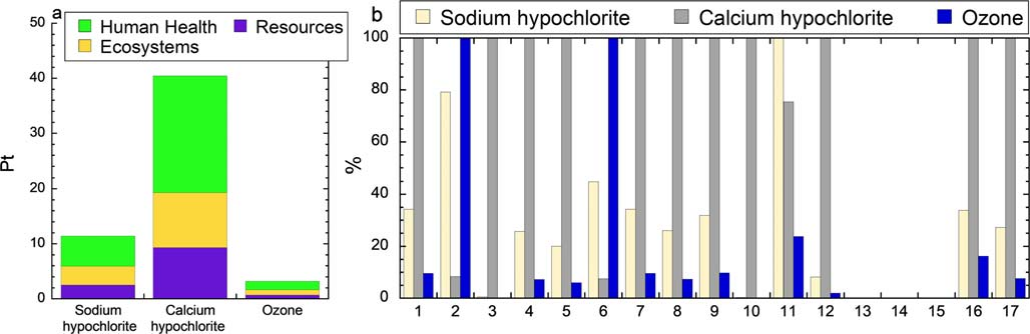
Environmental impact assessment of sodium hypochlorite, calcium hypochlorite, and ozone: single score (**a**) and characterization (**b**), where: 1—climate change human health; 2—ozone depletion; 3—human toxicity; 4—photochemical oxidant formation; 5—particulate matter formation; 6—ionizing radiation; 7—climate change ecosystems; 8—terrestrial acidification; 9—freshwater eutrophication; 10—terrestrial ecotoxicity; 11—freshwater ecotoxicity; 12—marine ecotoxicity; 13—agricultural land occupation; 14—urban land occupation; 15—natural land transformation; 16—metal depletion; 17—fossil depletion

Environmental impact assessment at life cycle stages (life cycle impact assessment—LCIA) was conducted on the different surface disinfection technologies of water supply facilities using sodium and calcium hypochlorite (150 mg/L active chlorine) and ozonated water (1 mg/L). As follows from the results of the inventory analysis (Figs. S3–S5, Tables S2–S4), the considered options for the different surface disinfection treatments of water supply facilities were characterized by environmental aspects such as the consumption of raw materials and energy, emissions of pollutants into the atmospheric air, wastewater discharges into water bodies or local sewer network, and waste generation. The lowest value of the eco-indicator (Dreyer et al. 2003, Goedkoop 1999) corresponded to the use of ozone (Fig. 6). Calcium hypochlorite had the highest impact on the environment among all but three impact categories (Fig. 6). Sodium hypochlorite had the highest impact in the freshwater ecotoxicity category. Ozone had the most detrimental environmental effect in the categories ozone depletion and ionizing radiation.

## Discussion

Worldwide, most surface disinfection treatments for water facilities rely on chlorine-containing disinfectants. A systematic choice of the best possible surface disinfectant should, beside their disinfection efficiency, include considerations such as environmental impact, risk for corrosion of water facilities (and thereby water quality and life time), and costs. The test conditions of this study were chosen based on common and recommended test conditions for surface disinfection treatments of water facilities with hypochlorite and ozone. Previous studies have shown that the efficiency of ozone treatment for 30 min is comparable or better than that of hypochlorite solution treatments for 24 h (Романовский et al. 2016).

### Comparison of surface disinfectants for water facilities—corrosion perspective

This study shows that, from a corrosion perspective, treatment with ozone is preferable over hypochlorite treatments. We base this statement on the following observations: (i) there is a lower corrosion rate during ozone treatment as compared with hypochlorite solutions, and (ii) there is no risk of trapping chlorine-containing corrosion products or hypochlorite species in pores and surface defects after the surface disinfection treatments.

The results of this study are limited by its boundary conditions, e.g., it is only valid at room temperature and for the active chlorine concentrations used. The pH was influenced by the hypochlorite concentration and not buffered, similar to what would be expected in water facilities. It is not totally clear from our results whether the pH or the hypochlorite concentration influences the corrosion rate to a greater extent, and most probably the effects seen are influenced by both parameters. The pH was found to affect the kinetics of open-circuit potential relaxation towards the steady state value, the steady state open-circuit potential value, and the amount of iron that was dissolved in solution. Despite the generally passivating effect of alkaline pH values for steel (Schmuki et al. 1999), the effect of increasing hypochlorite concentration, and thus higher solution pH, was found to result in increased corrosion as evident by weight loss measurements and corrosion product analysis.

There are at present relatively few studies related to corrosion of steel in hypochlorite solutions and dissolved ozone in media of relevance for ground water. Our study agrees with the conclusions of one study on mild steel in hypochlorite solution, showing that corrosion rates increase with freely available chlorine concentrations (Gaur et al. 1994). Our study, even though not directly comparable, is consistent with the conclusions of another study (Wu et al. 2014) on carbon steel that corroded more in the presence of ozone in air.

The relatively strongly decreased pH observed during hypochlorite exposure of steel could be explained by the consumption of OH^−^ ions according to the following reaction (Talaiekhozani et al. 2017):

$$ 2\ \mathrm{Fe}{\left(\mathrm{OH}\right)}_3+3\ {\mathrm{Cl}\mathrm{O}}^{-}+4\ {\mathrm{OH}}^{\hbox{--}}\to 2\ {\mathrm{FeO}}_4{2}^{-}+3{\mathrm{Cl}}^{-}+5\ {\mathrm{H}}_2\mathrm{O} $$

This is supported by the observation that no corresponding pH decrease was observed during pH reference measurements of hypochlorite solutions in the absence of steel (data not shown).

Further mechanistic insights in the corrosion of steel in hypochlorite containing and ozonated artificial tap water solutions are not possible from this study alone. However, it has been suggested in the literature that oxidation of mild steel in ozonated sea water results in the formation of a protective surface oxide due to the incorporation of calcium and magnesium ions in the surface film (Liao et al. 2012). This is supported by our observation that Ca-containing insoluble corrosion products formed increasingly with exposure time. This also suggests that the addition of inhibitors during ozone treatment could further reduce the corrosion rate of steel in water facilities, which should be investigated in future studies.

### Comparison of surface disinfectants for water facilities—environmental perspective

From an environmental perspective, the ozone treatment has an advantage over the hypochlorite treatments, as no/limited waste products are generated. Although ozone is classified in similar hazard classes as sodium hypochlorite regarding aquatic life (ECHA 2019a), it has the advantage that it can directly be generated in the water facility in a closed system and that its half lifetime is on average 20 min. This results in significantly lower impact on the environment of ozone treatment as compared with sodium or calcium hypochlorite treatments.

The highest environmental impact of calcium hypochlorite on the environment among the surface disinfectants can mainly be explained by the significant environmental impact during its production phase. It should also be noted that the preparation of a fresh surface disinfectant solution of calcium hypochlorite includes an initial preparation at 10 wt%, which then should be stored for 24 h, filtered from insoluble sediments, and finally be diluted to the needed concentration. In contrast, the production of sodium hypochlorite includes only electrolysis of sodium chloride on site. For the final surface disinfectant solution of sodium hypochlorite, this by-product solution is only diluted, without any formation of sediments. Ozone is generated and injected into the water immediately during the treatment.

Life cycle impact assessments of surface disinfection treatments of water facilities are scarce, while they are relatively common for water or wastewater disinfection treatments (Dong et al. 2017, Mo et al. 2018). In these studies, it was found that ozone has a lower environmental impact as compared with chlorine-containing disinfectants, which agrees with this study on surface disinfection.

### Comparison of surface disinfectants for water facilities—other aspects

Except life cycle impact assessment and lifetime of the material related to corrosion, important aspects to consider for surface disinfection of water facilities are the time, which in practice means a stop of the drinking water produced, and the easiness of operation. If it takes long time and requires handling of chemicals, flushing, and waste effluents, the surface disinfection treatment will be delayed more commonly and is less preferred. For these aspects, ozone requiring the shortest time of treatment (30 min) as compared with 1 day for chlorine-based treatments shows a clear advantage. It furthermore requires the least chemical and waste handling.

## Conclusions

This study compared steel corrosion and life cycle impact assessment of different surface disinfection treatments for internal surfaces of water facilities, such as boreholes, filters, pipes, and reservoirs, using hypochlorite-based and ozone-based processes. The following main conclusions were drawn:

1. The steel corroded actively in all solutions of pH 7.5 or higher; however, most severe corrosion occurred in the hypochlorite solutions of highest concentration.
2. The ozone treatment caused significantly less corrosion as compared with the sodium or calcium hypochlorite treatments with 150–250 mg/L active chlorine.
3. Hypochlorite or chlorine-containing compounds were trapped in corrosion products, defects, and cracks after the surface disinfection treatment with hypochlorite and were shown to influence the pH and open-circuit potential in subsequent exposure to artificial tap water. Trapped chlorine-containing compounds are likely to influence the corrosion of steel after surface disinfection treatments.
4. The amount of released and soluble iron to the tap water solutions was significantly lower (about 1%) as compared with the total oxidized mass of iron under given experimental conditions. This agreed with chemical speciation modeling.
5. Life cycle impact assessment revealed ozone to have the lowest negative impact on the environment, while calcium hypochlorite had the highest environmental impact due to its production phase.
6. In all, corrosion, life cycle impact assessment, and handling differences (preparation, handling, and waste) all prefer ozone for a surface disinfection treatment of water facilities.

## Electronic supplementary material

ESM 1 (PDF 1152 kb)

ESM 2 (DOCX 13 kb)
