# Supplementary material for: Comparison of different surface disinfection treatments of drinking water facilities from a corrosion and environmental perspective
Source: Environ Sci Pollut Res Int. 2020 Feb 1;27(11):12704–16. doi: 10.1007/s11356-020-07801-9 (PMC7136315; doi:10.1007/s11356-020-07801-9)
Supplement: Supplementary file 1 — (PDF 1152 kb) [file 11356_2020_7801_MOESM1_ESM.pdf]

Supplementary material – Environmental Science and Pollution Research

Comparison of different surface disinfection treatments of drinking water facilities from a corrosion and environmental perspective

Valentin Romanovski[1, 2, 3,], Per Martin Claesson[1, 4], Yolanda Hedberg[1,]**

[1]Division of Surface and Corrosion Science, Department of Chemistry, School of Engineering Sciences in Chemistry, Biotechnology and Health, KTH Royal Institute of Technology, SE-100 44, Stockholm, Sweden

[2]Center of Functional Nano-Ceramics, National University of Science and Technology "MISIS", 119049, Moscow, Russia

[3]Institute of General and Inorganic Chemistry, National Academy of Sciences of Belarus, 220072 Minsk, Belarus

[4]RISE Research Institutes of Sweden, Bioscience and Materials – Surface, Process and Formulation, SE-114 86 Stockholm, Sweden

*correspondence to: Valentin Romanovski, E-mail: vramano@kth.se, phone: +375295696874 or Yolanda Hedberg, E-mail: yolanda@kth.se, phone: +4687906878.

Content: Tables S1-S5, Figures S1-S11, additional text

1. Further details – materials and methods

1. 1. Justification of the steel type used for this study

A significant part of water supply structures and networks consists of metal parts, such as water wells, filters, pipelines, valves. Analysis by means of photometric method on a spark vacuum optical emission spectrometer (analyzer of metals and alloys) OBLF QSN 750 of some samples of steels taken from various water treatment facilities in Belarus (Brest region) is presented in Table S1.

Table S1. The average composition of the water well pipes by four samples in wt%

Sample	Fe	C	Si	Mn	Mo
1	balance	0.12	0.08	0.51	–
2	balance	0.14	0.06	0.51	–
3	balance	0.09	0.01	0.39	–
4	balance	0.12	0.07	0.49	–
5	balance	0.21	0.30	0.61	0.01
6	balance	0.07	0.26	0.45	–
7	balance	0.11	0.03	0.34	–
8	balance	0.14	0.04	0.29	–
9	balance	0.46	0.32	0.65	–
10	balance	0.10	0.22	0.43	–
11	balance	0.13	0.01	0.43	0.00

1. 2. Iron determination in solution and corrosion products

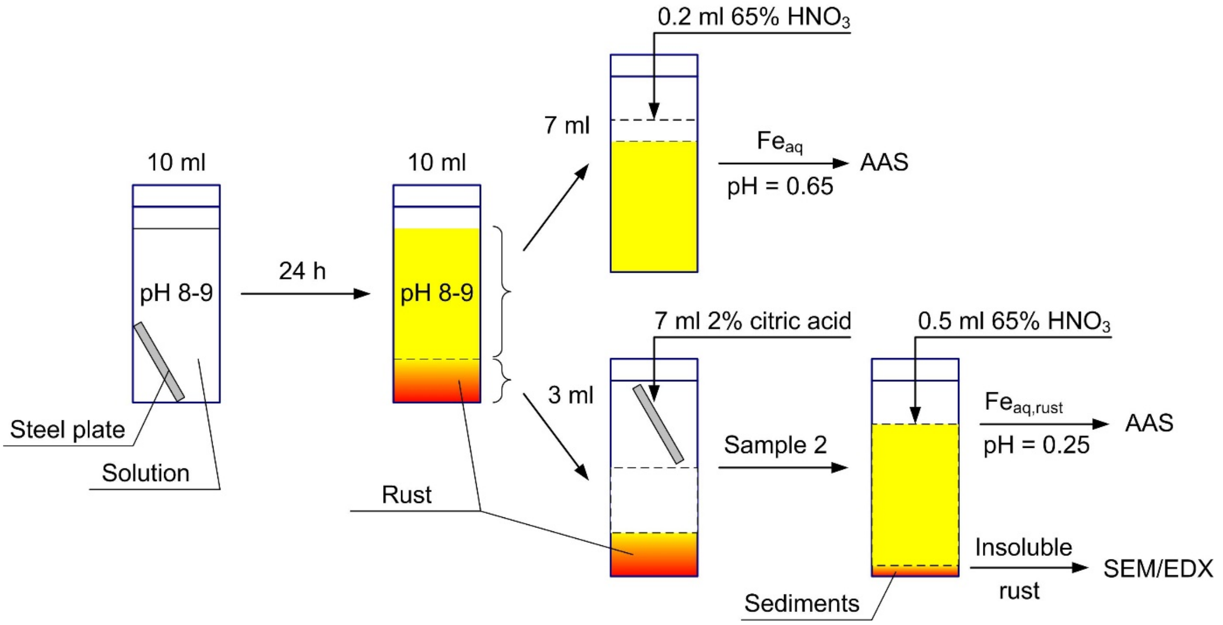

Fig. S1. The scheme of sample preparation for solution analysis of iron by means of AAS.

1. 3. Life cycle impact assessment

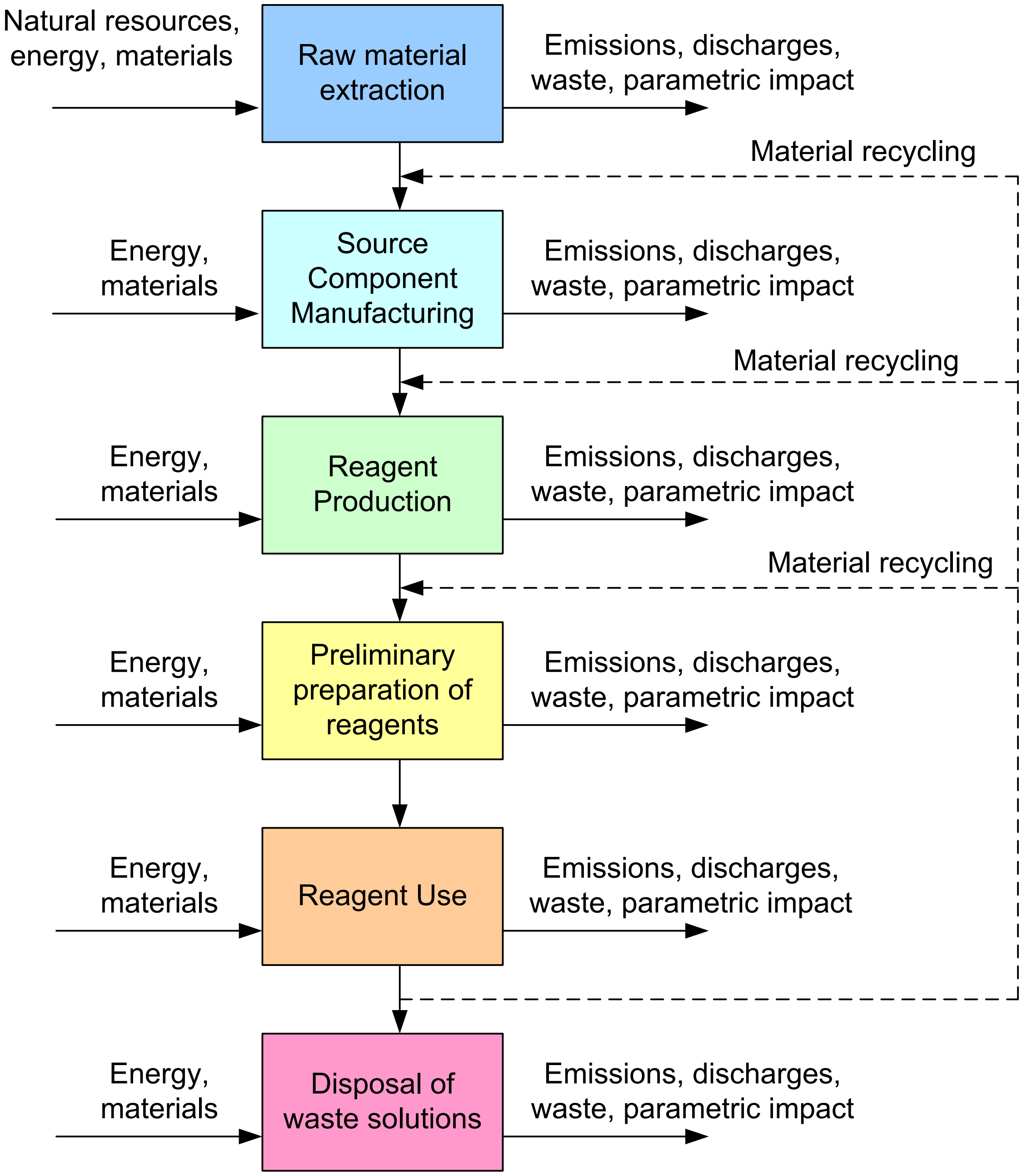

Fig. S2. General scheme of life cycle impact assessment

1. 3. 1. Assumptions and input parameters – sodium hypochlorite

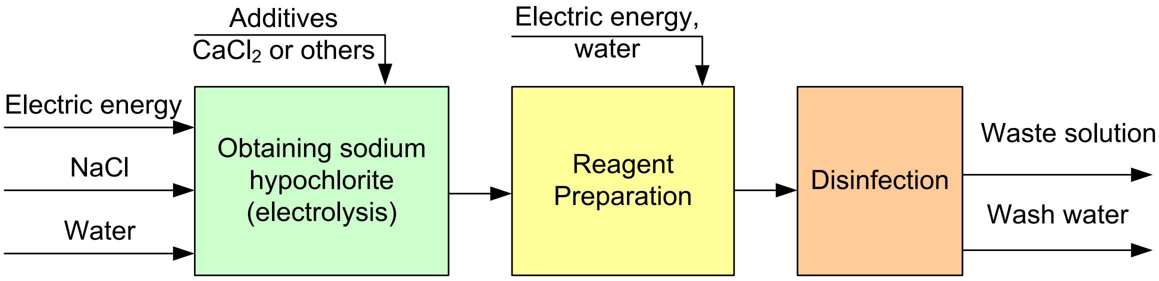

Fig. S3. Scheme of the life cycle of disinfection of water supply facilities with sodium hypochlorite solution

Table S2. The results of the inventory analysis of input and output flows using sodium hypochlorite solution for surface disinfection of water supply facilities

Stage	Input streams	Output streams	Impact on the environment
**Reagent Preparation**			
Obtaining of sodium hypochlorite	NaCl – 1420 g; water – 54 L; $CaCl_2$ – 14.2 g; electric energy – 94 kWh	Sodium hypochlorite solution – 18.8 L with mass concentration of active chlorine 0.8% (8 g/L); hydrogen – 0.27 L.	Hydrogen emissions
**Disinfection of water supply facilities**			
Preparation of sodium hypochlorite solution	sodium hypochlorite solution – 18.8 L; water – 981.2 L; electric energy – 1.5 kWh	Sodium hypochlorite solution with mass concentration of active chlorine 150 mg/L – 1 m^3	Wash water
The use of sodium hypochlorite solution	Sodium hypochlorite solution – 1 m^3; water – 2 m^3; electric energy – 1.5 kWh	Waste solution – 1 m^3; wash water – 2 m^3	Discharge of waste solution and wash water in the sewer network or on the terrain

1. 3. 2. Assumptions and input parameters – calcium hypochlorite

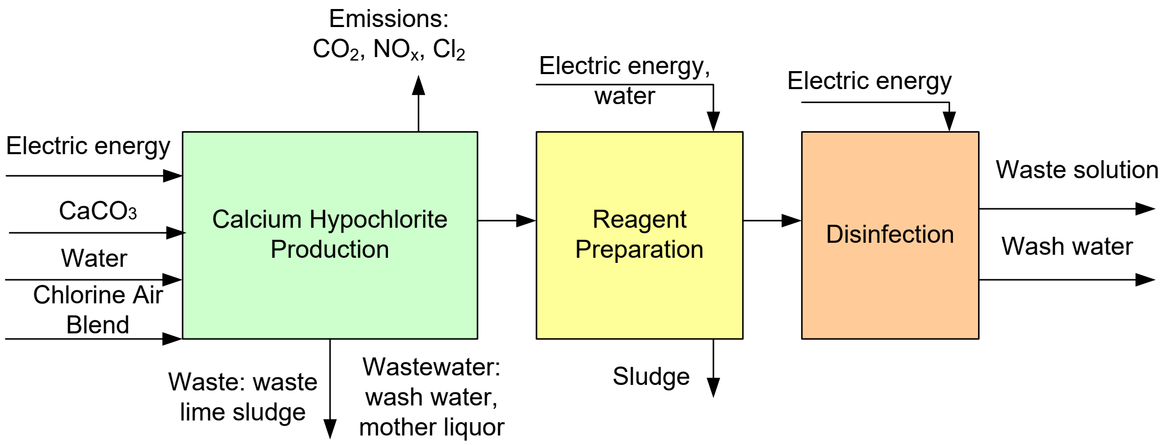

Fig. S4. Scheme of the life cycle of disinfection of water supply facilities with calcium hypochlorite solution

Table S3. The results of the inventory analysis of input and output flows using calcium hypochlorite solution for disinfection of water supply facilities

Stage	Input streams	Output streams	Impact on the environment
**Reagent Preparation**			
Calcium hypochlorite production	lime – 3767 g; water – 6020 g; electric energy – 15 kWh; Cl_2 – 370 g; air – 450 g	Calcium hypochlorite – 375 g with mass concentration of active chlorine 40%; H_2O_{vapor} – 109 g; CO_2 – 1344 g; inorganic dust – 344 g; CaO – 372 g; mother liquor (solution of $Ca(OH)_2$) – 536 g; Cl_2 – 0.16 g; NO_x – 0.04 g	Wastewater: wash water, mother liquor Waste: waste lime sludge Emissions: CO_2, NO_x, Cl_2
**Disinfection of water supply facilities**			
Preparation of calcium hypochlorite solution	Calcium Hypochlorite Solution – 375 g; water – 1 m^3; electric energy – 1.5 kWt	Calcium hypochlorite solution with active chlorine concentration 150 g/L – 1 m^3	Wash water
The use of calcium hypochlorite solution	Calcium Hypochlorite Solution – 1 m^3; water – 2 m^3; electric energy – 1.5 kWh	Waste solution – 1 m^3; wash water – 2 m^3	Discharge of waste solution and wash water in the sewer network or on the terrain

1. 3. 3. Assumptions and input parameters – ozone

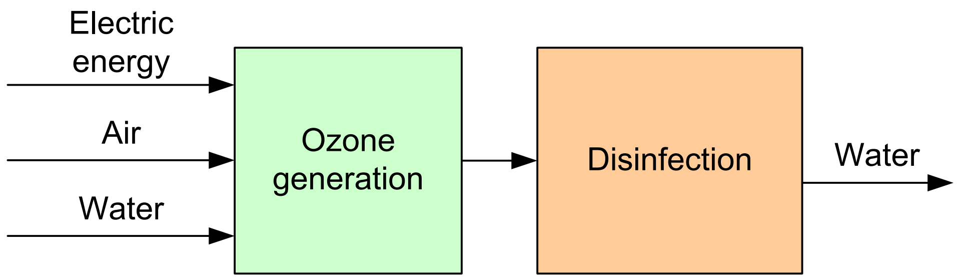

Fig. S5. Scheme of the life cycle of disinfection of water supply facilities with sodium hypochlorite solution

Table S4. The results of the inventory analysis of input and output flows using ozone solution for disinfection of water supply facilities

Stage	Input streams	Output streams	Impact on the environment
**Reagent preparation & disinfection of water supply facilities**			
Ozone generation	Air – 0.40 m^3; water – 1 m^3; electric energy –12 kWh (ozone generation and injection)	A solution of ozone in water with an ozone concentration of at least 1 mg/L – 1.0 m^3	–

2. Further details – results and discussion

2.1. Fraction of insoluble corrosion products as a function of time and disinfectant

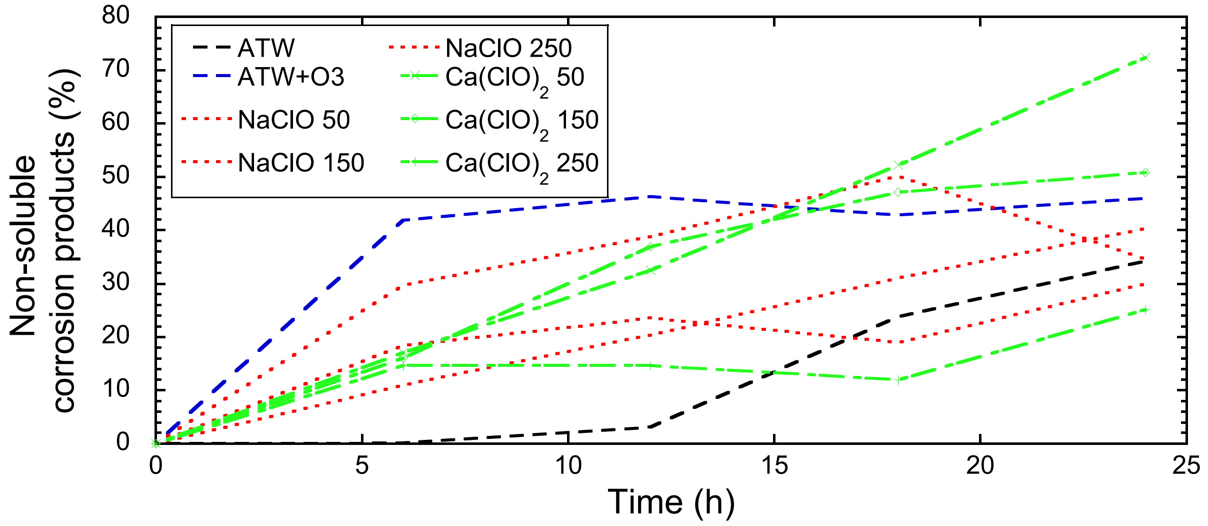

Fig. S6. Fraction of insoluble corrosion products in the citric and nitric acid procedure.

2.2. EDS analysis of insoluble corrosion products

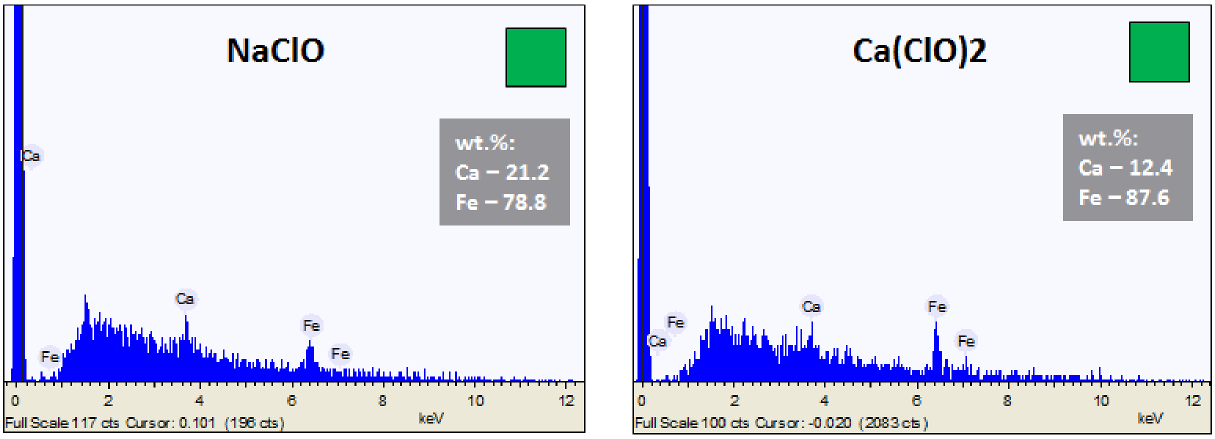

Fig. S7. Energy dispersive spectroscopy spectra of in nitric acid / citric acid (pH ≈ 0.25) insoluble rust sediments (c.f. Fig. S1)

2.3. Comparison of potentiodynamic polarization and weight loss

Faradays law

Faraday's law can be summarized by

$$m = \left(\frac{Q}{F}\right) \cdot \left(\frac{M}{z}\right)$$

where:

m – is the mass of the substance liberated at an electrode in grams

Q – is the total electric charge passed through the substance in Coulombs

F = 96500 C mol^{-1} is the Faraday constant

M – is the molar mass of the substance in grams per mol

z – is the valency number of ions of the substance (electrons transferred per ion).

In the simple case of constant-current electrolysis, $Q = I \cdot t$ leads to

$$m = \left(\frac{It}{F}\right) \cdot \left(\frac{M}{z}\right)$$

and then to

$$n = \left(\frac{It}{F}\right) \cdot \left(\frac{1}{z}\right)$$

where:

n – is the amount of substance ("number of moles") liberated: $n = m/M$

t – is the total time the constant current was applied.

The estimated weight loss from the potentiodynamic scan (for 15 min and linearly extrapolated to 6 hours) is shown in Fig. S8.

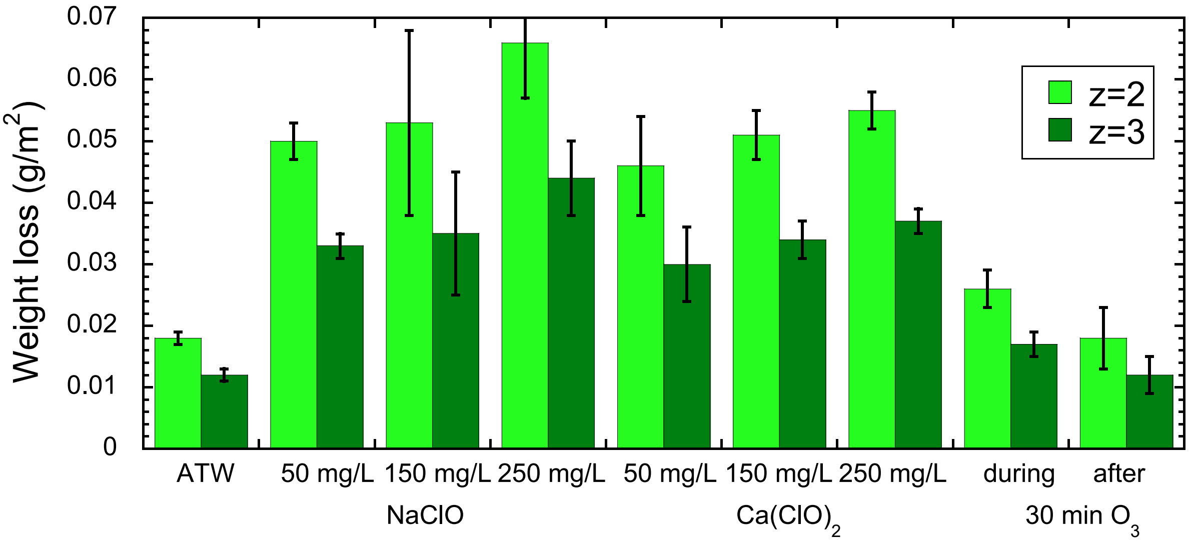

Fig. S8. Weight loss (g/m^2) extrapolated from the potentiodynamic polarization, calculated by Faraday's law for z=2 and for z=3 based on average figures of two independent measurements.

The ratio of weight loss calculated by Faraday's law for 6 h and results of weight loss measurements after 6 h are presented in Fig. S9.

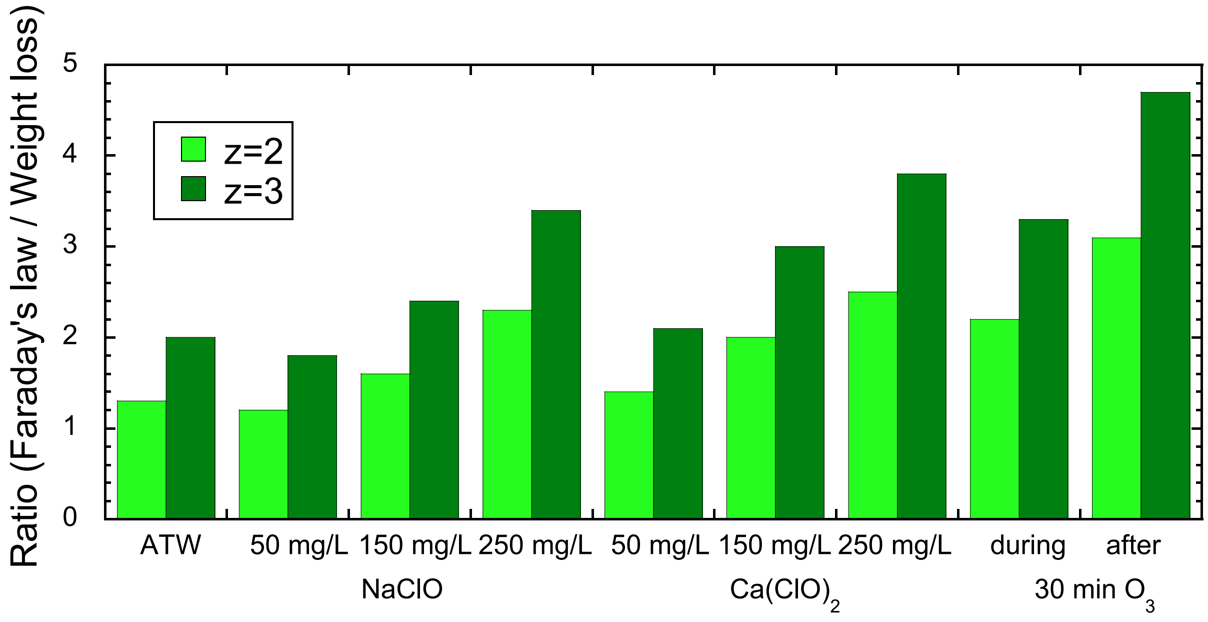

Fig. S9. Ratio of weight loss calculated by Faraday's law and weight loss measurements (6 h) for z=2 and for z=3 based on average figures of two measurements

2.4. Open-circuit potential

Figure S10 shows the open-circuit potential evolutions of the steel in artificial tap water adjusted to different pH values, artificial tap water treated by ozone (during first 30 or 45 min), artificial tap water with hypochlorite, and artificial tap water after a previous treatment with hypochlorite.

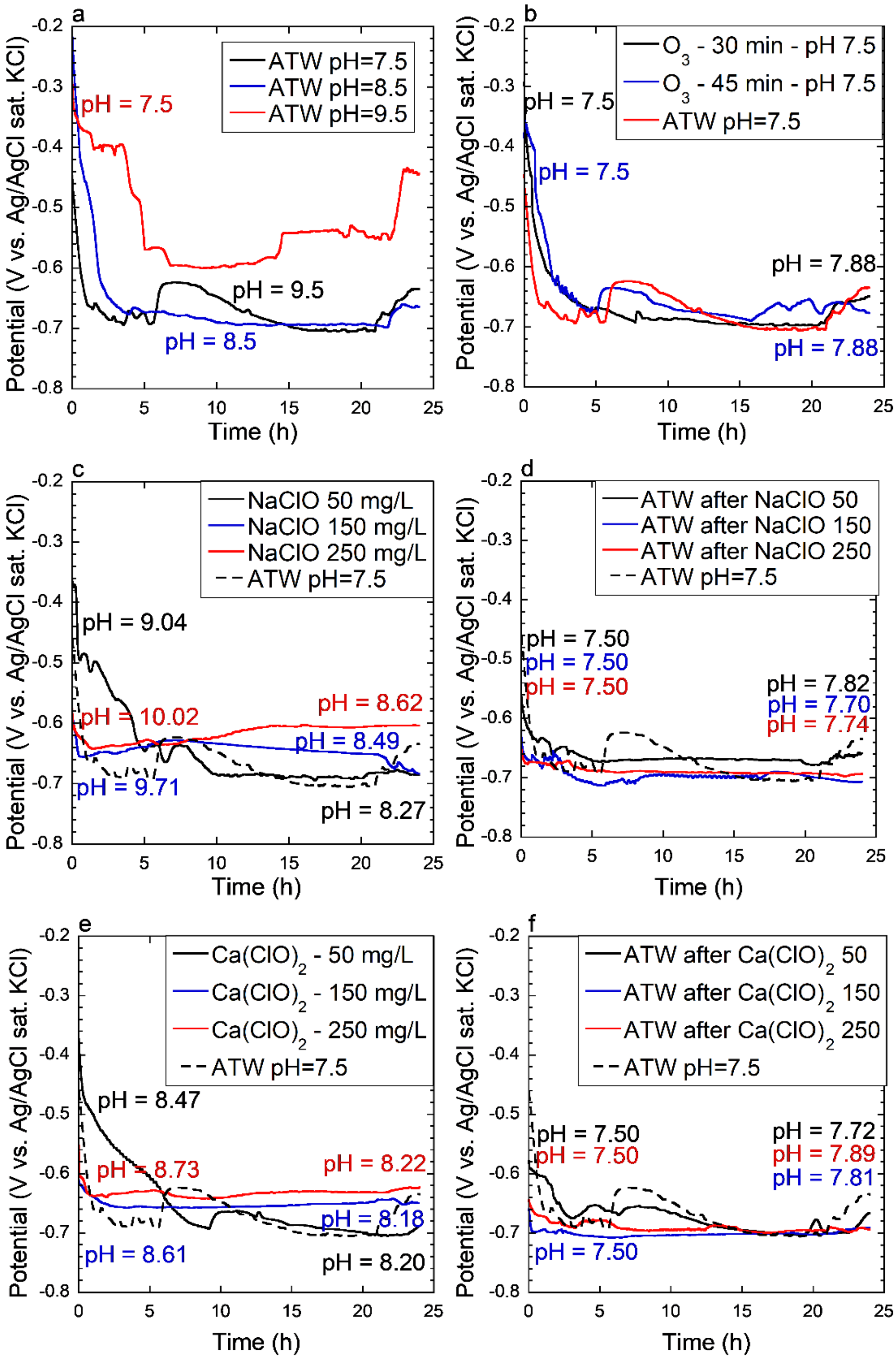

Fig. S10. The open-circuit potential evolution of the steel samples in artificial tap water (ATW) adjusted to different pH values **(a)**, artificial water treated by ozone during the first 30

or 45 min **(b)**, artificial tap water containing hypochlorite solutions **(c, e)**, and artificial tap water after previous treatment by hypochlorite solutions **(d, f)**. Average curves of two measurements are shown in all cases. The curve for artificial tap water at pH 7.5 is included for comparison in **b-f**. The initial and final pH values are included in figures **b-f**. $Ca(ClO)_2$ 50, 150, or 250 means 50, 150, or 250 mg/L active chlorine, respectively.

2.5. Elemental analysis of corrosion products after 24 h of treatment

Table S5 – Elemental analysis of corrosion products after 24 h treatment by means of EDS (wt%) – average data.

Solution	Fe	Cl	Na	Ca
Artificial water	100	*	*	*
Ozonated water	98.9	1.1	*	*
NaClO[a]	88.8	7.9	3.3	–
$Ca(ClO)_2$[a]	96.9	2.7	*	*

[a]250 mg/L active chlorine; * - less than 0.5 wt%

2.6. Single Raman spectra

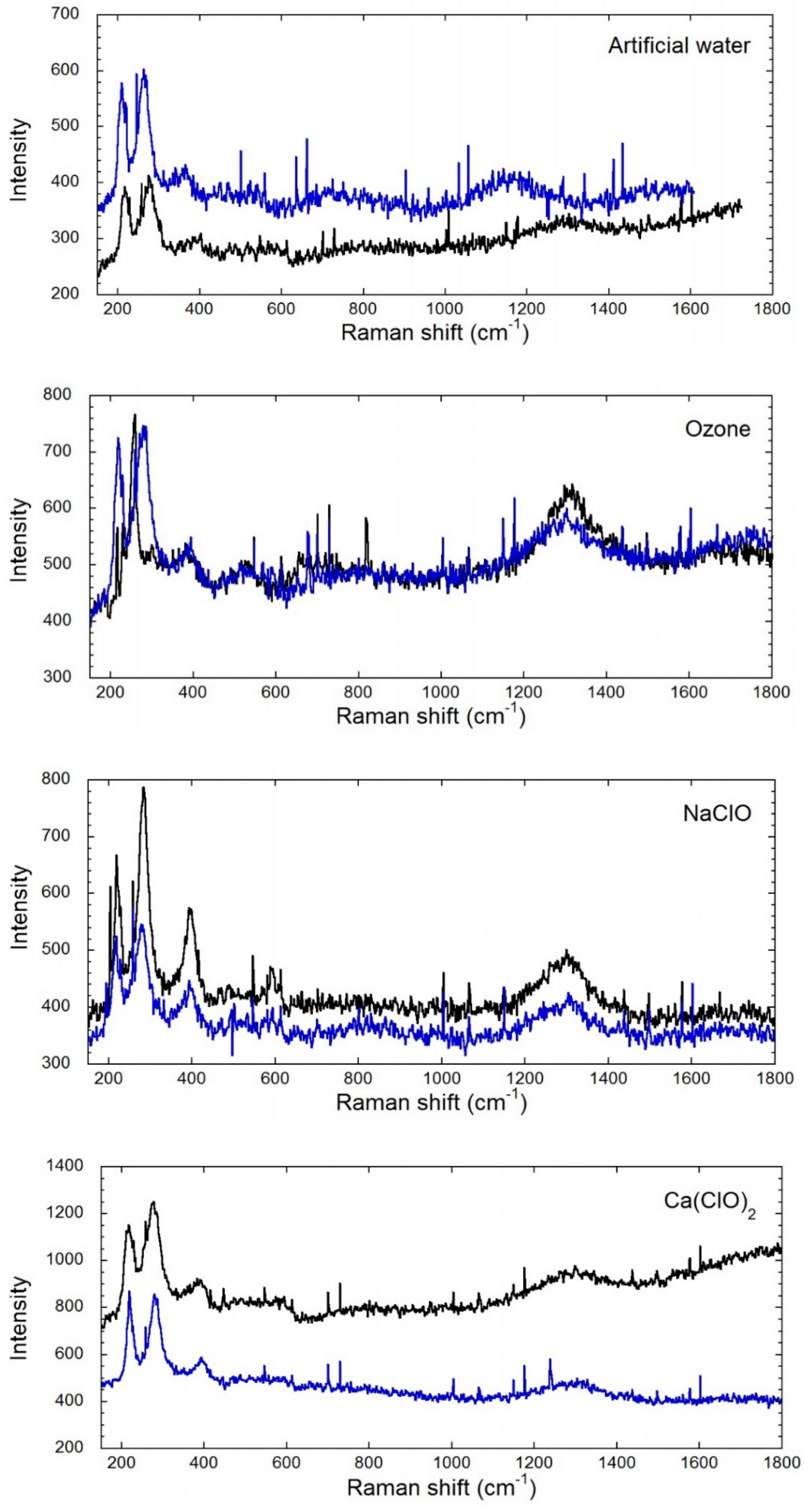

Fig. S11. Single Raman spectra of two replicates for each treatment.
