# Supplementary material for: Comparison of different surface disinfection treatments of drinking water facilities from a corrosion and environmental perspective
Source: Environ Sci Pollut Res Int. 2020 Feb 1;27(11):12704–16. doi: 10.1007/s11356-020-07801-9 (PMC7136315; doi:10.1007/s11356-020-07801-9)
Supplement: Supplementary file 2 — (DOCX 13 kb) [file 11356_2020_7801_MOESM2_ESM.docx]

**Supplementary information**

The supplementary information provides more detailed information about the steel composition, the life cycle impact assessment input values, the corrosion product solubility and composition, the raw data of figures of the main manuscript, and calculations of the transformed mass during corrosion measurements.
